# The “*L*-Sandwich” Strategy for True Coronary Bifurcation Lesions: A Randomized Clinical Trial

**DOI:** 10.1155/2023/6889836

**Published:** 2023-03-21

**Authors:** Quan Guo, Liang Peng, Lixin Rao, Cao Ma, Kang Zhao, Zhenzhou Zhao, Haiyu Tang, Muwei Li

**Affiliations:** ^1^Department of Cardiology, Henan Provincial People's Hospital, People's Hospital of Zhengzhou University, Zhengzhou, Henan, China; ^2^Department of Coronary Heart Disease of Central China Fuwai Hospital, Henan Key Laboratory for Coronary Heart Disease, Central China Fuwai of Zhengzhou University, Zhengzhou, Henan, China

## Abstract

**Background:**

This study explored the efficacy of the “*L*-sandwich” strategy, which involves the implantation of stents in the main vessel (MV) and shaft of the side branch (SB) with a drug-coated balloon (DCB) applied to the SB ostium, for coronary true bifurcation lesions.

**Methods and Results:**

Of 99 patients with true bifurcation lesions, 38 patients underwent the “*L*-sandwich” strategy (group *A*), 32 patients underwent a two-stent strategy (group *B*), and 29 patients underwent a single-stent + DCB strategy (group *C*). Angiography outcomes (late lumen loss [LLL], minimum lumen diameter [MLD]), and clinical outcomes (major adverse cardiac events [MACEs]) were analyzed. At 6 months, the MLD of the SB ostium in groups *A* and *B* were similar (*P* > 0.05) and group *A* larger than group *C* (*P* < 0.05). The LLL of group *B* was the largest among the three groups (*P* < 0.05). The MLD of the SB shaft in groups *A* and *B* were larger than in group *C* (*P* < 0.05). The LLL of the SB shaft in group *C* was the lowest (*P* < 0.05). Two patients in group *B* received target vessel revascularization at the 6-month followup (*P* > 0.05), and patients in the other groups had no MACEs.

**Conclusions:**

The “*L*-sandwich” strategy was feasible for the treatment of true coronary bifurcation lesions. It is a simpler procedure with similar acute lumen gain than the two-stent strategy, results in a larger SB lumen than the single-stent + DCB strategy, and it can also be used as a remedy for dissection following the single-stent + DCB strategy.

## 1. Introduction

Coronary bifurcation lesions account for about 15%–20% of total PCI [1], of which true bifurcation lesions account for 30%–40%. For the interventional treatment of true bifurcation lesions, the procedure is complicated and difficult and there are many intraoperative and postoperative complications that still represent a challenge for interventional cardiologists. In the two-stent strategy, due to the complexity of the anatomical structures and the limitations of imaging, poor attachment and underexpansion of the stent at the branch ostium, which causes in-stent thrombosis and restenosis, are common [2]. Delayed re-endothelialization may arise from multilayer stents, prolong the application time of dual antiplatelet drugs, and increase the risk of bleeding [3]. The single-stent strategy may cause displacement of the plaques and ridges of the branch ostium and thus insufficient blood flow to the branches [1]. Implanting a drug-coated balloon (DCB) in the branch seems to solve the shortcomings of double stenting [4]. However, there is a risk of hematoma or severe dissection with DCB implanted in a branch, which requires stent rescue [5], because the loss of some branches, such as the larger LCX, can be catastrophic. Corresponding author Prof. Li proposed a novel bifurcation strategy to circumvent the above problem, which involves the implantation of stents in the main vessel (MV) and the shaft of the side branch (SB) with a DCB applied to the ostium of the SB. Since this strategy resembles a “Sandwich” structure (stent-DCB-stent), and in order to distinguish the Sandwich strategy from other medical disciplines, we named it the “*L*-sandwich” strategy by combining the initials of Prof. Li's last name. In this study, we aimed to investigate the feasibility and efficacy of the “*L*-sandwich” strategy, with a view to improving the tedious operation process in true bifurcation lesions and reduce postoperative complications.

## 2. Methods

### 2.1. Study Population

This study complied with the Helsinki Declaration and was approved by the institutional ethics committee of Fuwai Central China Cardiovascular Hospital, Zhengzhou University (Zhengzhou, China). All participants in the study provided written informed consent. This trial has been registered on the ClinicalTrials website (NCT04753827).

**Figure 1:**
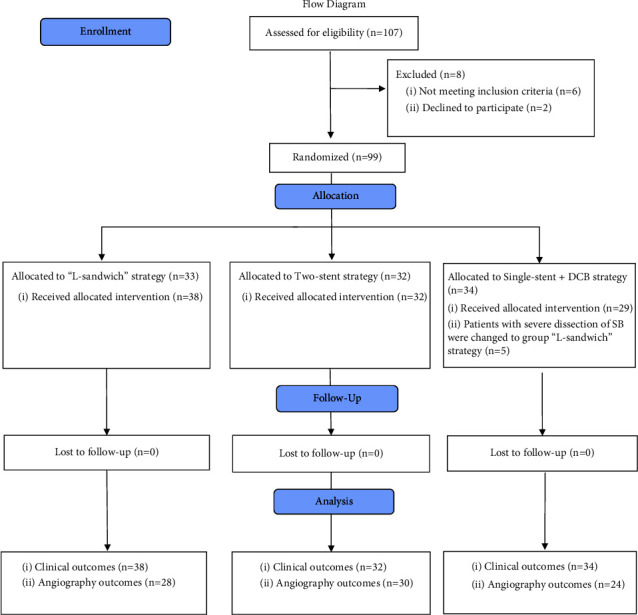
Research flow chart diagram.

This study was a single-center study where 107 patients were enrolled from Fuwai Central China Cardiovascular Hospital. Of these, 99 patients were randomized after screening for inclusion and exclusion criteria over the period from October 2021 to January 2022. Randomization was performed using a computer-generated list and SPSS software (IBM SPSS 25.0; SPSS Inc., Armonk, NY). Eighty-two patients underwent a 6-month angiographic followup (Figure 1). The inclusion criteria were: (1) true bifurcation disease (Medina classification: 1, 1, 1) and (2) SB diameter >2.5 mm and SB lesion length >25 mm. The exclusion criteria were: (1) the presence of cardiogenic shock or cardiopulmonary resuscitation, (2) acute myocardial infarction, (3) inability to tolerate dual antiplatelet therapy, (4) severe hepatic and renal insufficiency, and (5) life expectancy less than 1 year. Patients were divided into three groups according to different strategies. Group *A* underwent the “*L*-sandwich” strategy, whereby stents were implanted in the MV and the distal part of the SB, and approximately 3–5 mm was reserved at the SB ostium to apply a DCB. Group *B* underwent a two-stent strategy whereby stents were implanted in the MV and SB. Group *C* underwent a single-stent + DCB strategy whereby crossover MV stenting with SB DCB angioplasty were applied. When the SB in group *C* showed a type *C* or above dissection or hematoma (according to the National Heart, Lung and Blood Institute classification), or an SB thrombolysis in myocardial infarction flow grade <3 after predilation, the procedure was changed to that of group *A*.

### 2.2. PCI Procedure

**Figure 2:**
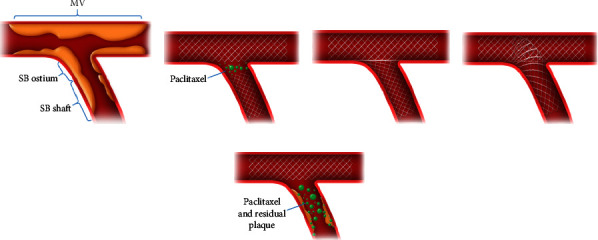
Panel (a) is a pattern diagram of a true bifurcation lesion, with measurement points marked. Panel (b) represents the “*L*-sandwich” strategy; panels (c) and (d) represent the DK-crush and culotte technique of the “two-stent” strategy; panel (e) represents the “single-stent + DCB” strategy. MV: main vessel; SB: side branch.

A radial artery approach was used in all patients. All stents were second-generationdrug-eluting stents and all the DCBs were SeQuent® Please (B. Braun, Melsungen, Germany). The size of semicompliant balloon, noncompliant (NC) balloon, cutting balloon, DCB, and stent was determined by the operator. The DCB was inflated within 120 s of entry into the body and inflated for 30–60 s. According to the current requirements, before DCB release, the following needs to be met: residual stenosis not greater than 30%, and no flow-limiting dissection (the processing strategy pattern diagram for different groups is shown in Figure 2).

### 2.3. Technical Details

In group *A*, predilation of the MV and SB was performed, followed by the placement of a stent on the shaft of the SB. After the SB shaft stent was postdilated by an NC balloon, the stent was implanted in the MV and postdilated using an NC balloon matching the diameter of the MV. A third guide wire was recrossed and the MV stent cell at the side branch ostium was adequately dilated using a cutting balloon. Afterwards, the DCB was deployed in the ostium of the SB, followed by kissing balloon inflation (KBI) using two NC balloons, and finally proximal optimization technique (POT) was performed on the MV (Supplementary Figure 1 for a more detailed step-by-step diagram).

In group *B*, after predilation, the stents were placed in the MV and SB (the operator decided to use a DK-Crush or Culotte technique), and the stents were postdilated by NC balloons with high-pressure, followed by final KBI and POT.

In group *C*, a stent was implanted in the MV, then fully postdilated by the NC balloon. Subsequently, the third guide wire was recrossed and the MV stent cell at the side branch ostium was predilated with a cutting balloon, and then a DCB was deployed in the ostium of the SB, followed by POT.

After percutaneous coronary intervention (PCI), all patients received dual antiplatelet therapy (DAPT) for at least 6 months. Secondary prevention medications were prescribed according to current guidelines and patient tolerance.

### 2.4. Followup

Clinical followup was performed by office visit or telephone at 30 days and every 3 months after discharge. Patients were advised to return for coronary angiography at 6 months ± 14 days.

### 2.5. Study Outcomes

The primary outcome was late lumen loss (LLL) of the MV and SB. The secondary outcomes were minimum lumen diameter (MLD) and acute gain and restenosis. In this study, we divided the SB into two parts for separate analysis. These parts were the “ostium,” which is about 3–5 mm from the beginning of the SB and the “shaft,” which is about 3–5 mm away from the ostium part. In terms of in-hospital and long-term clinical outcomes; we focused on major adverse cardiac events (MACEs: acute myocardial infarction, cardiac death, target vessel revascularization).

### 2.6. Quantitative Coronary Analysis (QCA)

QCA was analyzed using Cardiovascular Angiographic Analysis System (CAAS) II software version 5.0 (Pie Medical Imaging, Maastricht, the Netherlands). Analysis was performed by two cardiologists separately. At followup, restenosis was defined as a QCA diameter stenosis ≥50%. The analysis was performed independently by two physicians blinded to the group, and the results were averaged.

### 2.7. Statistical Analysis

Baseline characteristics are reported as counts and percentages or mean ± standard deviation (SD). The chi-square test was used to compare categorical variables. Comparison of means between multiple groups was performed using analysis of variance (one-way ANOVA) followed by Bonferroni's posthoc tests. All statistical analyses were performed using SPSS software v25.0 (IBM, Armonk, NY, USA). *P* values were two-tailed, and a *P* value of <0.05 was considered statistically significant. Power calculations were performed using PASS software version 15. According to previous pre-experimental results, a total of 60 patients were needed to detect a difference in the LLL of 0.2 mm with a power of 90% and a two-sided 5% significance level.

## 3. Results

### 3.1. Baseline and Procedural Characteristics

A total of 99 patients were recruited and randomized, resulting in 33 in group *A*, 32 in group *B*, and 34 in group *C*. In group *C*, five patients with SB dissection were changed to group *A*. Finally, there were 38 people in group *A*, 32 in group *B*, and 29 in group *C*. There was no significant difference in terms of sex, age, hyperlipidemia, hypertension, diabetes, smoking, body mass index (BMI), and left ventricular ejection fraction (LVEF) among the three groups (Table 1).

Procedural characteristics are shown in Table 2. There was no statistical difference between the three groups in pre-PCI angiography characteristics. Post-PCI, there was no statistical difference in the MLD of the MV between the three groups (3.31 ± 0.41 mm vs. 3.29 ± 0.19 mm vs. 3.37 ± 0.36 mm, *P*=0.594). The MLD and diameter acute gain of the SB ostium in group *B* were significantly larger than that in group *A*, and in group *A* than that of group *C* (MLD: 2.42 ± 0.14 mm (*A*) vs. 2.77 ± 0.15 mm (*B*) vs. 2.24 ± 0.16 mm (*C*), *P* < 0.05; acute gain: 1.76 ± 0.14 mm (*A*) vs. 2.07 ± 0.12 mm (*B*) vs. 1.57 ± 0.21 mm (*C*), *P* < 0.05). For the SB shaft, the MLD and diameter acute gain in groups *A* and *B* were larger than that in group *C*, but there was no significant difference between group *A* and group *B* (MLD: 2.73 ± 0.13 mm (*A*) vs. 2.70 ± 0.14 mm (*B*) vs. 2.27 ± 0.15 mm (*C*), *P* < 0.05; acute gain: 2.00 ± 0.10 mm (*A*) vs. 1.93 ± 0.12 mm (*B*) vs. 1.53 ± 0.18 mm (*C*), *P* < 0.05). Group *C* was the best in terms of procedural time and contrast agent dosage, followed by group *A*, then group *B* (procedural time: 79.29 ± 11.83 min (*A*) vs. 105.72 ± 11.64 min (*B*) vs. 64.69 ± 11.88 min (*C*), *P* < 0.05; contrast volume: 59.37 ± 9.54 ml (*A*) vs. 90.25 ± 19.11 ml (*B*) vs. 50.69 ± 10.81 ml (*C*), *P* < 0.05).

### 3.2. Clinical Outcomes

Two patients in group *B* received target vessel revascularization at the 6 months after the procedure due to in-stent restenosis, and patients in the other groups had no MACEs, but there was no significant difference among the groups (*P*=0.118) (Table 3).

### 3.3. Angiography Followup

**Figure 3:**
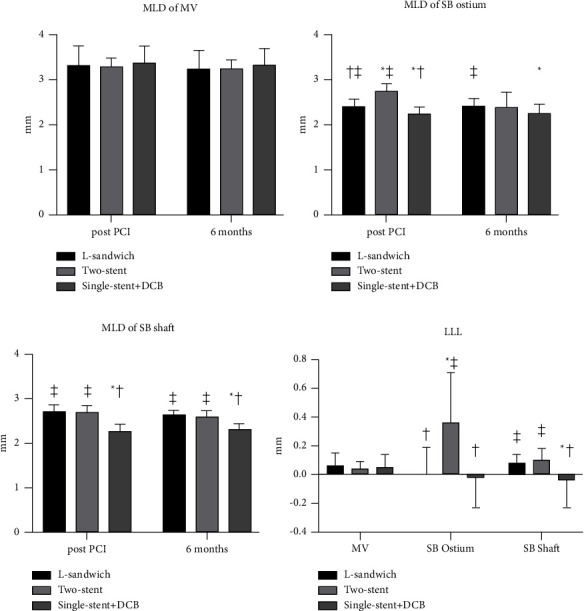
Statistically significant compared to the “*L*-sandwich” group; ^†^statistically significant compared to the two-stent group; ^‡^statistically significant compared to the single-stent + DCB group. Panels (a)–(c) show MV, SB ostium, and SB shaft, respectively, and the comparison of the MLD in each group at post-PCI and 6 months. Panel (d): compare LLL in three groups at 6 months. MLD: minimum lumen diameter; MV: main vessel; SB: side branch; DCB: drug-coated balloon; LLL: late lumen loss.

Followup angiography after 6 months was performed in 82.83% (82/99) of patients (Table 4 and Figure 3). The MLD of the MV showed no statistical difference between the groups (3.25 ± 0.40 mm vs. 3.24 ± 0.21 mm vs. 3.33 ± 0.37 mm, *P*=0.541). In terms of the MLD of the SB ostium, at the time of followup, there was no significant difference between group *A* and group *B*, but group *A* was greater than group *C* (2.43 ± 0.15 mm (*A*) vs. 2.40 ± 0.33 mm (*B*) vs. 2.25 ± 0.21 mm (*C*), *P* < 0.05), while group *B* had significantly greater LLL (0.00 ± 0.19 mm (*A*) vs. 0.36 ± 0.35 mm (*B*) vs. −0.02 ± 0.21 mm (*C*), *P* < 0.05). The MLD of SB shaft in group *A* and group *B* was still significantly larger than that in group *C* (2.63 ± 0.11 mm (*A*) vs. 2.59 ± 0.14 mm (*B*) vs. 2.31 ± 0.12 mm (*C*), *P* < 0.05). However, group *C* had the lowest SB shaft diameter LLL (0.08 ± 0.06 mm (*A*) vs. 0.10 ± 0.08 mm (*B*) vs. −0.04 ± 0.19 mm (*C*), *P* < 0.05).

## 4. Discussion

This study evaluated the clinical and angiographic outcomes of a new strategy we proposed to deal with the true coronary bifurcation lesions. The major findings were: (1) the “*L*-sandwich” strategy is feasible as a treatment for true bifurcation lesions, or as a remedy for SB severe dissection following the single-stent strategy; (2) compared with the two-stent strategy, the “*L*-sandwich” strategy resulted in nearly the same late lumen diameter at the SB ostium, and it also had better acute gain than the single-stent + DCB strategy; and (3) the “*L*-sandwich” strategy was simpler to perform than the two-stent strategy, with less X-ray exposure dose and contrast agent use.

Mainstream strategies have their pros and cons. The two-stent strategy can solve the stenosis of the SB ostium caused by plaque shift and carina shift followed by MV stent implantation, and the immediate effect is better than the single-stent strategy [6]. However, the operation of double stenting is complicated and difficult for novices, and no matter what technique is used, there is a certain degree of LLL [7]. This is consistent with the results of our study, whereby the MLD of the SB ostium in group *B* decreased after 6 months compared with post-PCI, while both groups *A* and *C* were almost unchanged. Group *B* required the most contrast use and X-ray exposure.

Herrador et al. [8] and the PEPCAD V study [4] showed the long-term advantages of DCB in the treatment of bifurcation lesions, reporting that the single-stent + DCB strategy has a lower incidence of MACE and better prognosis than two-stent. Unfortunately, in the single-stent strategy, after the MV is implanted with a stent, the bifurcated plaque is squeezed to the branch, which affects the lumen area at the SB ostium, resulting in aggravation of angina symptoms in some patients after PCI. Moreover, transporting a long bail-out stent through the MV stent cells may be difficult or inaccurate positioning when SB occurs flow-limiting dissection or hematoma in single-stent + DCB strategy, and this may lead to increased adverse events. This proportion reached approximately 15% in this study, suggesting that the single-stent + DCB strategy is not very safe. In addition, the acute lumen benefit of SB shafts using DCB is not as good as stent implantation because it is currently accepted that DCB residual stenosis only needs to be less than 30% [9].

The “*L*-sandwich” strategy has special advantages for the SB, especially for patients with longer lesions and severe SB dissections. The “*L*-sandwich” strategy implants a stent in the SB shaft so that the acute gain of the diameter can be similar to the two-stent technique and dissection can be avoided. When SB dissection occurs during the single-stent strategy, the “*L*-sandwich” strategy is also a remedy to prevent the further development of dissection or intermural hematoma. The “*L*-sandwich” strategy reserves a 3–5 mm stent-free area at the SB ostium to avoid stent overlapping and stent deformation at the bifurcation after the two-stent strategy, thereby reducing the incidence of stent thrombosis and restenosis [2, 10]. In the stent-free area between the two stents, a cutting balloon or NC balloon with the same diameter as the SB is used for high-pressure dilatation, and then the DCB is expanded, avoiding the “geographic misplacement” of the stent in the two-stent technique. DCB delivers the drug to the vessel wall quickly and evenly, leaving no metal or polymer in the vessel, does not affect the normal contraction and diastolic function of the blood vessel, does not affect the repair of the endothelium, and without the risk of advanced stent thrombosis [11]. In addition, DCB has a positive remodeling effect [12], which is consistent with our study: the LLL of lesions used DCB is almost zero, and there is even a late lumen diameter gain. The higher pressure of the dilate SB ostium lumen further reduces the incidence of restenosis [13].

The “*L*-sandwich” strategy also simplifies the operation process, shortens operation time and reduces the amount of contrast agent and radiation compared to the two-stent strategy. It is an easy-to-learn operation technique.

### 4.1. Study Limitations

This study was a single-center study with a small sample size, which reduces the generalizability of the results. The next step is to perform a multi-center randomized controlled study to provide more evidence of the feasibility of this technique. Intracoronary imaging (intravascular ultrasound or optical coherence tomography) was not performed in most patients due to the high cost, and therefore, data related to the lumen area could not be obtained. However, the benefits of the “*L*-sandwich” strategy were clearly demonstrated by coronary angiography. We will continue to follow these patients to observe longer-term clinical outcomes.

## 5. Conclusion

The “*L*-sandwich” strategy is feasible for the treatment of true coronary bifurcation lesions, and particularly more beneficial for the SB ostium. It not only results in better SB shaft acute lumen gain than the single-stent strategy but also shows nearly the same late lumen diameter at the SB ostium as the two-stent strategy. The success rate is higher than that of the single-stent + DCB strategy, and the steps are simpler than the two-stent strategy. This technique should be considered, especially when the SB is long, the lesions are severe, or with flow-limiting dissection.

## Figures and Tables

**Table 1:** Clinical baseline characteristics of groups.

	“L-sandwich” (*n* = 38)	Two-stent (*n* = 32)	Single-stent + DCB (*n* = 29)	*P*
Male [*n*, %]	30 (78.9)	25 (78.1)	23 (79.3)	0.993
Age (years)	60.76 ± 10.51	56.78 ± 13.40	62.34 ± 12.96	0.186
Hyperlipidemia [*n* (%)]	29 (76.3)	20 (62.5)	22 (75.9)	0.371
Hypertension [*n* (%)]	17 (44.7)	17 (53.1)	20 (69.0)	0.140
Diabetes [*n* (%)]	16 (42.1)	10 (31.3)	6 (20.7)	0.176
Smoking [*n* (%)]	18 (47.4)	9 (28.1)	13 (44.8)	0.223
BMI	25.41 ± 1.83	25.37 ± 2.07	25.47 ± 2.05	0.924
LVEF (%)	59.50 ± 5.94	61.00 ± 6.63	60.93 ± 5.13	0.494

Values are mean ± SD or *n* (%). BMI, body mass index; LVEF: left ventricular eject fraction.

**Table 2:** Lesion and procedural characteristics at pre- and post-PCI.

		“L-sandwich” (*n* = 38)	Two-stent (*n* = 32)	Single-stent + DCB (*n* = 29)	*P*
Lesion location (*n*)					
LM-LCX		8	13	6	0.579
LAD-D		17	11	13	
LCX-OM		10	5	7	
RCA-PLA/PDA		3	3	3	
Bifurcation angle (degrees)		62.58 ± 17.15	56.78 ± 12.90	55.83 ± 10.97	0.106
Main vessel					
Reference diameter (mm)		3.37 ± 0.41	3.36 ± 0.19	3.43 ± 0.35	0.652
Target lesion length (mm)		22.56 ± 6.25	20.81 ± 6.43	20.79 ± 7.6	0.452
Stent length (mm)		26.05 ± 6.33	25.83 ± 6.21	25.69 ± 7.76	0.866
MLD (mm)	Pre-PCI	0.91 ± 0.22	0.89 ± 0.26	0.95 ± 0.23	0.533
	Post-PCI	3.31 ± 0.41	3.29 ± 0.19	3.37 ± 0.36	0.594
Acute gain (mm)		2.39 ± 0.33	2.40 ± 0.34	2.42 ± 0.33	0.944
Residual stenosis (%)		1.83 ± 1.90	2.11 ± 1.93	1.78 ± 1.64	0.730
Side branch					
Reference diameter (mm)		2.97 ± 0.13	2.92 ± 0.14	2.92 ± 0.11	0.207
Target lesion length (mm)		28.67 ± 3.32	29.59 ± 3.72	28.80 ± 4.45	0.572
Cutting balloon [*n*, %]		38(100)	2(6.3)	29(100)	<0.001
Stent length (mm)		24.40 ± 3.61	34.13 ± 2.85		<0.001
DCB length (mm)		15.00 ± 0.00		32.37 ± 3.82	<0.001
Ostium MLD (mm)	Pre-PCI	0.65 ± 0.09	0.70 ± 0.09	0.66 ± 0.11	0.113
	Post-PCI	2.42 ± 0.14	2.77 ± 0.15	2.24 ± 0.16	^*∗*^<0.001
					^†^<0.001
					^‡^<0.001
Ostium acute gain (mm)		1.76 ± 0.14	2.07 ± 0.12	1.57 ± 0.21	^*∗*^<0.001
					^†^<0.001
					^‡^<0.001
Ostium residual stenosis (%)		18.50 ± 4.50	5.01 ± 1.53	23.44 ± 5.34	^*∗*^<0.001
					^†^<0.001
					^‡^<0.001
Shaft MLD (mm)	Pre-PCI	0.74 ± 0.08	0.77 ± 0.10	0.73 ± 0.13	0.272
	Post-PCI	2.73 ± 0.13	2.70 ± 0.14	2.27 ± 0.15	^*∗*^0.908
					^†^<0.001
					^‡^<0.001
Shaft acute gain(mm)		2.00 ± 0.10	1.93 ± 0.12	1.53 ± 0.18	^*∗*^0.117
					^†^<0.001
					^‡^<0.001
Shaft residual stenosis (%)		7.87 ± 1.38	7.50 ± 1.65	22.4 ± 5.11	^*∗*^1.000
					^†^<0.001
					^‡^<0.001
POT [*n* (%)]		36 (94.7)	32 (100)	27 (93.1)	0.349
Procedural time (min)		79.29 ± 11.83	105.72 ± 11.64	64.69 ± 11.88	^*∗*^<0.001
					^†^<0.001
					^‡^<0.001
Contrast volume (ml)		59.37 ± 9.54	90.25 ± 19.11	50.69 ± 10.81	^*∗*^<0.001
					^†^<0.001
					^‡^0.035

^*∗*^: “*L*-sandwich” strategy vs. two-stent strategy; ^†^: two-stent strategy vs. single-stent + DCB strategy, ^‡^: “*L*-sandwich” strategy vs. single-stent + DCB strategy, bonferroni's post hoc test. *D*: diagonal artery; DCB: drug-coated balloon; LAD: left anterior descending coronary; LCX: left circumflex coronary artery; MLD: minimum lumen diameter; OM: obtuse marginal artery; PDA: posterior descending branch; PLA: posterior of left ventricle artery; RCA: right coronary artery; PCI: percutaneous coronary intervention; POT: proximal optimization technique.

**Table 3:** Clinical outcomes.

	“L-sandwich” (*n* = 38)	Two-stent (*n* = 32)	Single-stent + DCB (*n* = 29)	*P*
TVR [*n*, %]	0	0	2 (6.3%)	0.118
AMI	0	0	0	—
Cardiac death	0	0	0	—

TVR: target vessel revascularization; AMI: acute myocardial infarction.

**Table 4:** Angiography follow-up outcomes.

		“*L*-sandwich” (*n* = 28)	Two-stent (*n* = 30)	Single-stent + DCB (*n* = 24)	*P*
Main vessel					
MLD (mm)	Post-PCI	3.32 ± 0.43	3.29 ± 0.19	3.38 ± 0.37	0.659
	6 months	3.25 ± 0.40	3.25 ± 0.19	3.33 ± 0.37	0.602
LLL (mm)		0.06 ± 0.09	0.04 ± 0.08	0.05 ± 0.09	0.625
Side branch ostium					
MLD (mm)	Post-PCI	2.42 ± 0.15	2.76 ± 0.15	2.23 ± 0.17	^*∗*^<0.001
					^†^<0.001
					^‡^<0.001
	6 months	2.43 ± 0.15	2.40 ± 0.33	2.25 ± 0.21	^*∗*^1.000
					^†^0.074
					^‡^0.033
LLL (mm)		0.00 ± 0.19	0.36 ± 0.35	−0.02 ± 0.21	^*∗*^<0.001
					^†^<0.001
					^‡^0.818
Side branch shaft					
MLD (mm)	Post-PCI	2.71 ± 0.14	2.69 ± 0.15	2.27 ± 0.15	^*∗*^0.698
					^†^<0.001
					^‡^<0.001
	6 months	2.63 ± 0.11	2.59 ± 0.14	2.31 ± 0.12	^*∗*^0.679
					^†^<0.001
					^‡^<0.001
LLL (mm)		0.08 ± 0.06	0.10 ± 0.08	−0.04 ± 0.19	^*∗*^1.000
					^†^0.002
					^‡^<0.001

^*∗*^: “*L*-sandwich” strategy vs. two-stent strategy; ^†^: two-stent strategy vs. single-stent + DCB strategy, ^‡^: “*L*-sandwich” strategy vs. single-stent + DCB strategy, bonferroni's post hoc test. MLD: minimum lumen diameter; PCI: percutaneous coronary intervention; LLL: late lumen loss.

## Data Availability

The data used to support the findings of this study are available from the corresponding author upon request.

## Supplementary Materials

Supplementary Materials Supplementary Figure 1: step-by-step diagram of the “*L*-sandwich” strategy. Panel (A) Pattern diagram of a true bifurcation lesion; Panel (B) Side Branch (SB) dilatation using a compliant balloon after placement of guide wires in the main branch (MV) and SB, respectively; Panel (C) MV dilatation using a compliant balloon; Panel (D) stent implantation at 3–5 mm from the branch ostium and post-dilation using a non-compliant (NC) balloon; Panel (E) stent implantation in the MV and post-dilation using a NC balloon; Panel (F) guide wire delivery through the MV stent cell to the SB; Panel (G) cutting balloon to dilate the MV stent cell and fully pre-dilate the SB ostium; Panel (H) drug-coated balloon implanted at the SB ostium; Panel (I) kissing balloon inflation using two NC balloons; Panel (J) proximal optimization technique of the MV using NC balloon; Panel (K) final result of “*L*-sandwich” strategy.
