# Supplementary figures and images for: The “L-Sandwich” Strategy for True Coronary Bifurcation Lesions: A Randomized Clinical Trial
Source: J Interv Cardiol. 2023 Mar 21;2023:6889836. doi: 10.1155/2023/6889836 (PMC10049845; doi:10.1155/2023/6889836)

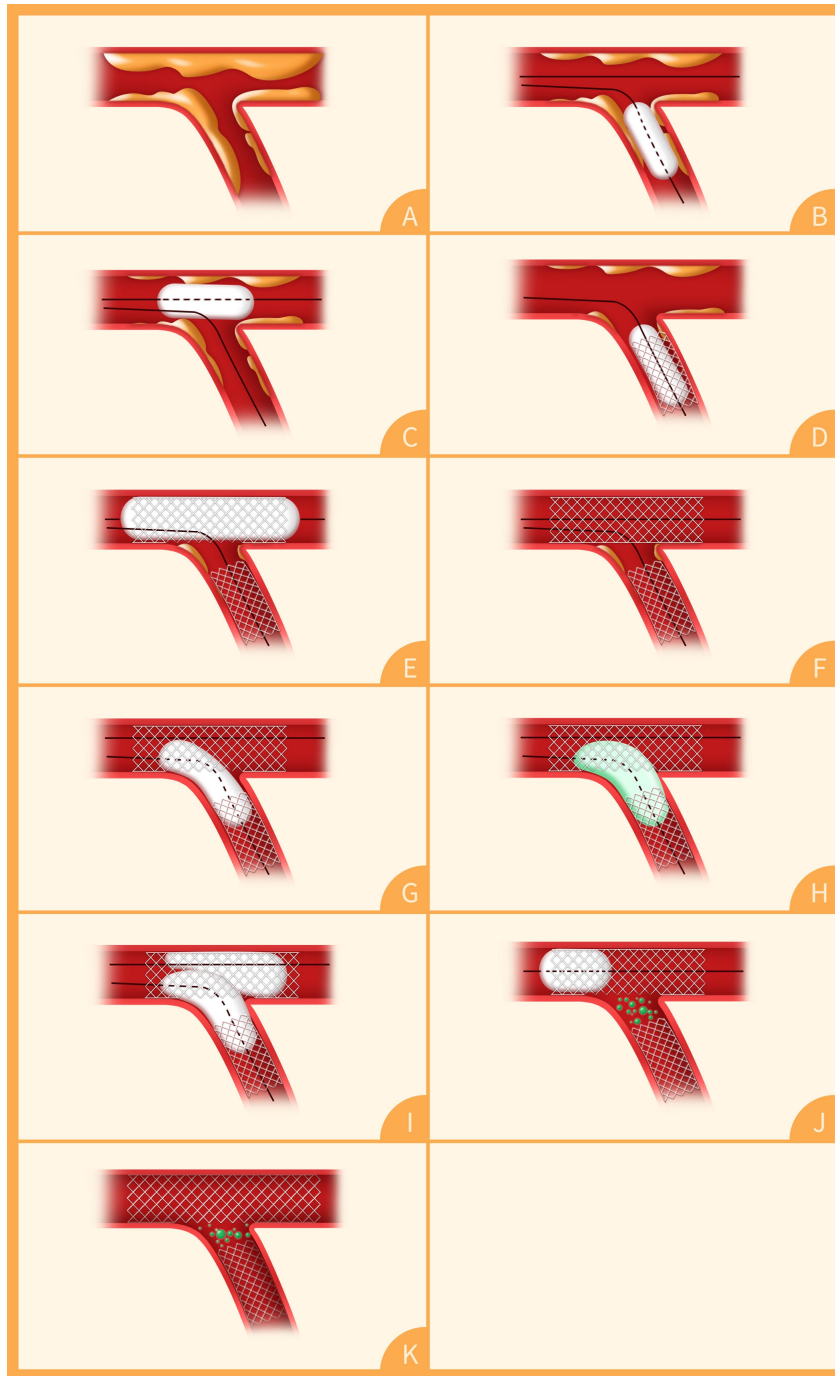

Supplement: Supplementary Materials — Supplementary Figure 1: step-by-step diagram of the “L-sandwich” strategy. Panel (A) Pattern diagram of a true bifurcation lesion; Panel (B) Side Branch (SB) dilatation using a compliant balloon after placement of guide wires in the main branch (MV) and SB, respectively; Panel (C) MV dilatation using a compliant balloon; Panel (D) stent implantation at 3–5 mm from the branch ostium and post-dilation using a non-compliant (NC) balloon; Panel (E) stent implantation in the MV and post-dilation using a NC balloon; Panel (F) guide wire delivery through the MV stent cell to the SB; Panel (G) cutting balloon to dilate the MV stent cell and fully pre-dilate the SB ostium; Panel (H) drug-coated balloon implanted at the SB ostium; Panel (I) kissing balloon inflation using two NC balloons; Panel (J) proximal optimization technique of the MV using NC balloon; Panel (K) final result of “L-sandwich” strategy. [file 6889836.f1.pdf]
